# Post-abortion needs-based education via the WeChat platform to lessen fear and encourage effective contraception: a post-abortion care service intervention-controlled trial

**DOI:** 10.1186/s12905-024-03004-3

**Published:** 2024-03-05

**Authors:** Danfeng Shi, Chenyin Liu, Lingna Huang, Xiao-Qian Chen

**Affiliations:** Fujian Provincial Maternal and Child Health Hospital, Fujian Fuzhou, China

**Keywords:** Temination of pregnancy, Post-abortion care, Needs-based education, WeChat group

## Abstract

**Objective:**

Our study aims to investigate post-abortion needs-based education via the WeChat platform for women who had intended abortion in the first trimester, whether they are using effective contraception or becoming pregnant again.

**Design:**

This single hospital intervention-controlled trial used a nearly 1:1 allocation ratio. Women who had intended abortions were randomly assigned to a Wechat group (needs-based education) and a control group (Traditional education). The women's ability to use effective contraception was the main result. Whether they unknowingly became pregnant again was the second result. Another result was patient anxiousness. Before and after education, women filled out questionnaires to assess their contraception methods and anxiety.

**Methods:**

Based on the theoretical framework of contraceptions of IBL (inquiry-based learning), post-abortion women were included in WeChat groups. We use WeChat Group Announcement, regularly sending health education information, one-on-one answers to questions, and consultation methods to explore the possibilities and advantages of WeChat health education for women after abortion. A knowledge paradigm for post-abortion health education was established: From November 2021 until December 2021, 180 women who had an unintended pregnancy and undergone an induced or medical abortion were recruited, their progress was tracked for four months, and the PAC service team monitored the women's speech, discussed and classified the speech entries and summarized the common post-abortion needs in 8 aspects. At least 2 research group members routinely extracted records and categorized the outcomes.

**Results:**

Before education, there were no appreciable variations between the two groups regarding sociodemographic characteristics, obstetrical conditions, abortion rates, or methods of contraception (*P* > 0.05). Following education, the WeChat group had a greater rate of effective contraception (63.0%) than the control group (28.6%), and their SAS score dropped statistically more than that of the control group (*P* < 0.05). Following the education, there were no unwanted pregnancies in the WeChat group, whereas there were 2 in the traditional PAC group. Only 5 participants in the WeChat group and 32 in the conventional PAC group reported mild anxiety after the education.

## Introduction

Post-abortion care(PAC), launched by the global health community in the early 90 s of the last century, is a global initiative to provide quality gender and reproductive health services, and its implementation has become one of the key strategies to improve womens health and promote reproductive rights around the world [1]. Since then, PAC has been implemented and studied in many parts of the world, and some results have been achieved. A large number of women who have been consulted by PAC have benefited [2–4]. PAC has also been widely promoted and implemented in China, and our family planning team has been working since 2011 to provide PAC services to women with unintended pregnancies, encouraging the implementation of highly effective contraceptive measures to reduce the harm caused by further unintended pregnancies and repeated miscarriages. PAC has been recognized by more and more family planning service providers and researchers as a measure to ensure reproductive health and encourage women to take effective, and long-acting reversible contraception [5, 6]. This measure has reduced the rate of induced abortion and repeated termination of unintended pregnancies to a certain extent and improved the level of female reproductive health and mental health [7–9]. Although the study suggest that we justify our study based on expanding reproductive autonomy rather than on reducing unintended pregnancy and as a reproductive option to which all women should have access, [10], post-abortion complications caused by widespread miscarriage should not be ignored [11].

Many studies have shown that health education, psychological care, and humanistic care in the form of telephone communication and text messages after abortion can improve the physical and mental health of women after the intended abortion [12–15], and contraception provides a series of other potential benefits in addition to health, and some studies believe that it is necessary to promote various forms of health education models. Studies have indicated many benefits and possibilities of using mobile communication education methods for continuity services [16–18]. Moreover, services for women who have had unwanted pregnancies have been limited during the Covid-19 pandemic. In the context of the rapid development of the Internet and the popularization of mobile phone WeChat today, requiring access to medical help via telehealth and requiring self-managed abortion are the key findings of the identified studies. Women request termination of pregnancy and are satisfied with remote abortion care due to the flexibility and ongoing telephone support available [19]. The purpose of this research is to explore the application effect of the PAC service model based on the WeChat platform and the post-abortion needs of women with early termination of pregnancy, so as to improve the effective contraceptive measures after abortion, avoid unintended pregnancy and bad emotions again [20, 21]. Although a desired and planned pregnancy is associated with apparent benefits to the patient, an undesired or unplanned pregnancy may not be. Patients may choose to avoid pregnancy either altogether to optimize their health or postpone pregnancy such that pregnancy-related complications are less likely. Therefore, the benefit of choosing contraception vs. pregnancy will differ according to the patient's health and reproductive goals [22].

## Methods

### Participants and procedures

**Fig. 1:**
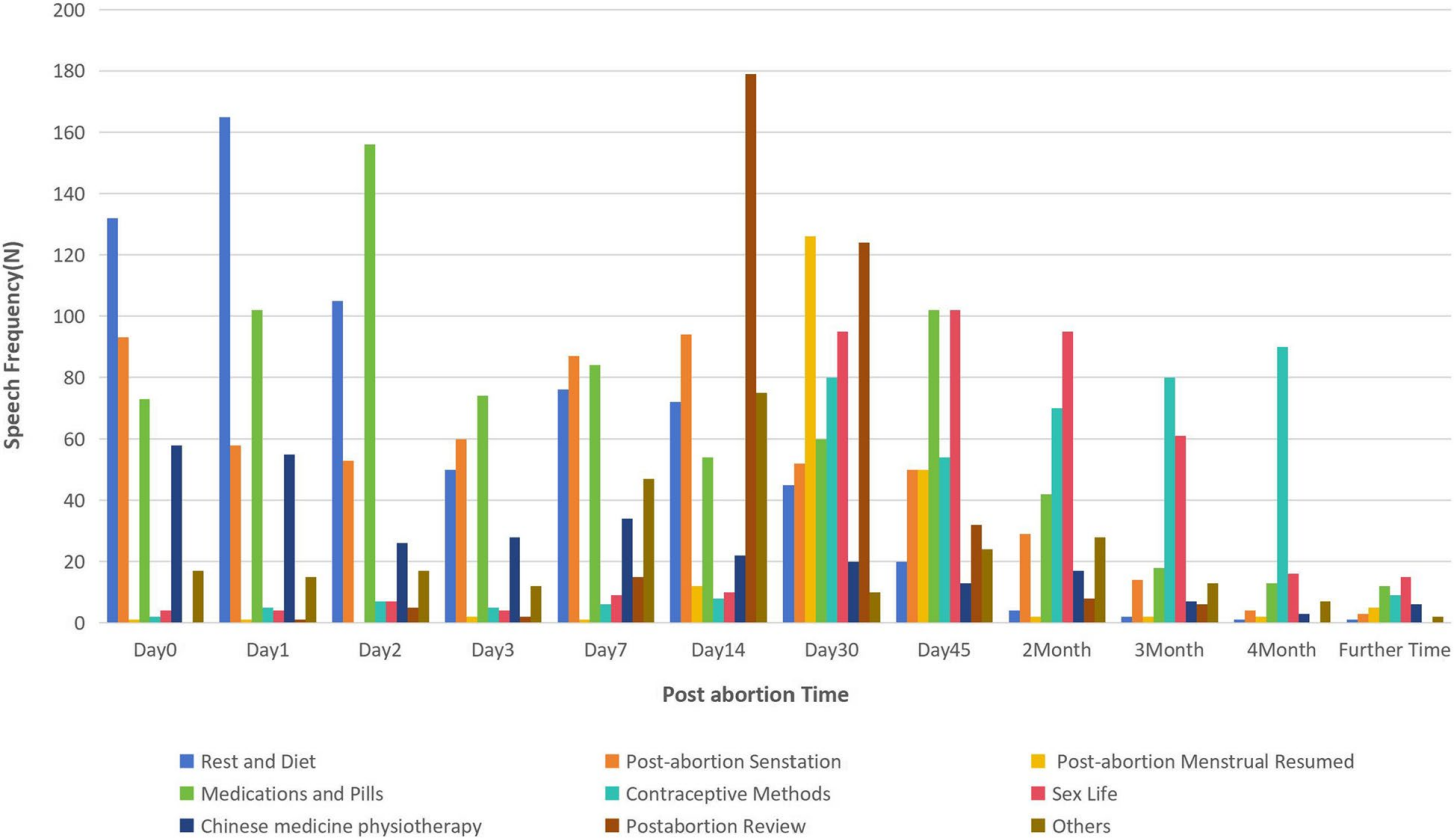
The early stage post-abortion requirements of 180 individuals

The PAC service team is made up of 1 family planning physician, 1 head nurse, and 2 charge nurses. Based on the theoretical framework, contraception methods of IBL ( inquiry-based learning) [23, 24] include post-abortion women in WeChat groups. We use WeChat Group Announcement WeChat dialogue, regularly send health education information, one-on-one answers to questions, and consultation methods to explore the possibilities and advantages of WeChat health education for women after abortion and show results according to the women's phased needs. Building a knowledge paradigm for post-abortion health education: From November 2021 until December 2021, 180 women who had an unwanted pregnancy and undergone an induced or medical abortion were a part of the research group, which tracked their progress four months after the procedure. The PAC service team monitored the patient's speech. At least 2 members of the research group discussed and classified the speech entries, summarized the common post-abortion needs of women in 9 aspects as an outline, routinely extracted records, and categorized the outcomes. The outcomes are then categorized into Rest and Diet. Medications and Pills, Physiotherapy using traditional Chinese medicine, Post-abortion different feelings, Contraception, Post-abortion menstruation transfer, Sex life, Post-abortion Review and Others. Equivalent to the period that women spend recovering from an abortion. The density and frequency of post-abortion concerns are finally summarized when each woman has the same type of project on their mind daily, and the frequency of speech for the day is calculated once. Using the information from the WeChat group speech summary, It has been discovered that patients' problems in the first week following the intended abortion are mainly focused on rest, diet, medication, and various physical sensations, while women in the second week after abortion pay attention more to rehabilitation-related issues like post-abortion blood loss volume and bleeding time; Women concentrate on the post-abortion menstruation transition one to one and a half months following the abortion. Create a post-abortion health education material appropriate for the post-abortion time and health needs based on demand and the frequency of queries [23] (Fig. 1).

After removing 6 initial questions based on age and menopausal status from each of the two groups of women who got 400 questionnaires, a final total of 394 initial questionnaires with an effective rate of 98.5% were obtained, including 198 instances in the WeChat group and 196 cases in the control group. This controlled-random trial was conducted in a single tertiary hospital collection for mother and child healthcare in a major city in southeast China. The health care unit was distinguished by being a clinic that provided integrated care in obstetric care and gynecological areas to women with a higher socioeconomic level intervention-controlled trial with a nearly 1:1 allocation ratio. The unit was attached to a medical university and served the population attributed to its surrounding area. The population number used for the sample calculation was 394 women who had undesired pregnancies between January and April 2022.

### Sample size calculation

According to the pre-experiment, the implementation rate of effective contraception was 50% in the intervention group and 33% in the control group, with a bilateral α = 0.05 and a power β of 90% [25]. The formula is calculated according to the sample size of the control trial.$n=\frac{{2\overline{p} \overline{q} \left({z}_{\alpha }+{z}_{\beta }\right)}^{2}}{{\left({p}_{1}-{p}_{2}\right)}^{2}}$. The calculation was as follows: *n* = 172, considering the 1:1 group, that is, 172 subjects in each of the intervention group and the control group, and considering a 15% loss to follow-up and rejection, the final number of participants in at least 198 intervention group and control group was 198 in the end. The follow-up process is described as follows, and 2 cases were lost in the control group.

As a result, 394 pregnant women made up the sample, and each of them had a one-on-one interview lasting roughly five minutes. 198 intended abortion women from Monday to Wednesday were assigned to either a Wechat group (Phased needs-based education) or a control group (Traditional education) from Thursday to Friday, with 196 women assigned to the control group. Women's more effective contraception was the primary outcome. The possibility that they would become pregnant again unintentionally was the secondary outcome. Another outcome was the women's anxiety. Women filled out questionnaires before and after receiving information to assess the choice of contraception and their anxiety women had to be between 18 and 40, with menopause occurring between 34 and 77 days. Either induced abortion or oral medical abortion for unplanned pregnancies; no fertility needs within a year, and willing participants who had no fertility restrictions. Because of the woman's unwanted pregnancy in the outpatient section, the intended abortion(including induced abortion and oral pregnancy medications) was chosen. Women who were illiterate and unable to complete the questionnaire independently, as well as those with psychiatric conditions and those unable to independently utilize the WeChat chat feature on a mobile device, were excluded from the study. The study procedure was described to patients after they consented to participate. All participants gave their consent in writing after being fully informed.

### Educations

By collecting the contents of the needs of women in each period (see Fig. 1) of the WeChat health education group in the early stage, women were informed about the goal and importance of joining the post-abortion care WeChat group based on the PAC service by the intervention group (WeChat group), which executed PAC services based on the WeChat platform and the staged needs of women. They were incorporated into the intended abortion treatment PAC services established by the study team's WeChat group based on the patients' staged needs, and 180 postoperative women helped assemble the important demands and difficulties of post-abortion women. Create health education queries that take post-abortion requirements and time into account (Fig. 1) and provide health information and psychological support in the WeChat group on the day of the intended abortion, 1 day to 14 days after the intended abortion, 15 to 30 days, 1 month to 3 month, until 6 months of post-abortion [12, 14–16, 18]. Meanwhile, control group women were also followed at the same time; the contents were the same, but the methods were different. They follow up by telephone or clinic on the post-abortion day, 14 days, 1 month,3 months, and 6 months (Table 1). A questionnaire including sociodemographic, obstetric, abortion, and contraceptive techniques and SASSASZung1971 (Self-Rating Anxiety Scale, 20 questions) scores before education was employed to examine two groups of unintended pregnancies. The corresponding health education was carried out in the WeChat intervention group by means of oral, WeChat group announcements, and WeChat dialogues. The controlled group provides PAC services on intended abortion day, including educating women about the risks and potential complications of abortion before the intended abortion, emphasizing the effects of repeated abortion on long-term fertility and the results of subsequent pregnancies, outlining post-abortion precautions, and urging women to choose post-abortion contraceptive methods, tell the women to come back to the clinic if she feels any unwell, and making telephone calls, post-abortion blood loss, whether menstruation resumes, whether sexual life resumes and the contraceptive methods used each time; encourage women who use less effective contraception to switch to effective methods [26–29]correctly guide and adhere to use; find out whether they are pregnant unintentionally again. The wechat group women ask specific physical and psychological questions in the group, and the researchers' doctors and nurses respond individually and offer consultations every day. At the same time, group members are encouraged to connect and communicate, and ladies who actively and accurately respond to questions are given praise and positive affirmation. Encourage group members to choose long-acting reversible contraception, intra-uterine devices (IUD), and implants as the efficacy is not dependent on compliance [29] and adhere to them, timely publicize the harm of abortion, provide positive counsel on reproductive health information when group members engage, and maintain the WeChat group for six months following an abortion.

**Table 1:** Education content in WeChat groups and control group

Post-abortion	Wechat group Contents	Control group
Time	Method	Method
**Day 0**	Jeopardization: both induced abortion and medical abortion, although a remedy for unwanted pregnancies, can cause harm to women. Short-term injuries are vaginal bleeding, incomplete miscarriage, long-term harm causing menstrual abnormalities, uterine adhesions, and secondary infertility. Rest: Get enough rest and sleep. Diet: establish good eating habits. Do not miss a meal. Take regular nutritious meals. Provide high protein, high-calorie beverages, nutritious finger foods, juice, and snacks as tolerated except ice spicy and stimulating foods. Do not overeat as a way of reducing stress.Medications: Take medications as ordered and observe for effectiveness and side effects. Teach about estrogen and progestin pills and continue to replenish pills promptly when missing.Contraception: Individualized contraceptive counseling for immediately implementation of long-acting reversible contraception used.Group Announcement, One-on-one consultation	Jeopardization: both induced abortion and medical abortion, although a remedy for unwanted pregnancies, can cause harm towomen. Short-term injuries are vaginal bleeding, incomplete miscarriage, long-term harm causing menstrual abnormalities, uterine adhesions,and secondary infertility.Rest: Get enough rest and sleep.Diet: establish good eating habits. Do not miss a meal. Take regular nutritious meals. Provide high protein, high-calorie beverages, nutritious finger foods, juice, and snacks as tolerated except ice spicy and stimulating foods. Do not overeat as a way of reducing stress. Medications: Take medications as ordered and observe for effectiveness and side effects. Teach about pills and continue to replenish pills promptly when missing. Contraception: Individualized contraceptive counseling for immediate implementation of long-acting reversible contraceptive misuse, One-on-one consultation
**Day1-Day14**	Exercise: including walking, gardening, and playing as tolerated, select mild and diversionary activities instead of stimulating exercise and competitive games in the top 3days post-abortion.7days post-abortion later develop an exercise routine. The Personal hygiene: Pay attention to physical needs for food, sleep, and hygiene. Prepare a warm shower and hot beverages to aid.Psychology: budget properly within the limits of your financial resources. Develop close relationships with family and community members, experiencing and expressing feeling. Help women to recognize and express feelings (denial, hopelessness, anger, guilt, blame, helplessness)Symptoms: normal of a small amount of vaginal bleeding within 10 days.Group Announcement, One-on-one consultation if possible	In the 14 days post-abortion, ultrasound re-examines the recovery of the uterus. Guides the use of contraceptives. Urges the effective contraception methods used if the sexual life resumes, Clinic
**Day15-Day30**	Diets: Principles of nutrition foods for wellness maintenance, water per day. Take regular nutritious meals. Contact a dietitian if weight gain becomes a problem. Review: Reminder of uterine ultrasound review.Contraception: Give guidance on contraceptive methods in person according to the patient's age and fertility history.Note: sexual intercourse is prohibited before menstruation.Psychology: seek opportunities to voice out your feelings and be involved in decisions.Get organization: Highly effective contraception should be implemented after abortion.Time management: Plan the work and use the time effectively to manage it. Do not do the same thing every day (if possible) Activities: The team approach uses the milieu to meet each person's needs.Knowledge: Emphasize the importance of monthly blood levels. Teach symptoms of Uterine adhesions or disseminate, Group Announcement, Speech in the group, One-on-one consultation if possible.	In the 1month post-abortion day followed up post-abortion condition: blood loss amount, whether menstruation resumes, whether sexual life resumes and the contraceptive methods used each time. Urge women who use less effective contraception change to effective contraception methods. Correctly guide and adhere to use.Find out whether they are pregnant unintentionally again.Answer women’s questions, Telephone, clinic
**1Mth-3Mths**	Pills: Healthy women can take estrogen and progestin pills continuously for more than 6 months, self observe for side effects. Teach about pills and continue to replenish pills promptly when missing. Contraception: Help with resolving difficulties related to IUD pattern choice and choosing a long-acting reversible contraception methods, encourage group solutions to daily living health problems. Use a kind, understanding, but emotionally neutral approach. Repeat simple, direct information. Assist in daily decisions.Knowledge: Emphasize the importance of monthly blood levels. Teach symptoms of Uterine adhesions or disseminate.Relaxing: take long hot baths, spend time alone, or periodically nap, Group Announcement, Speech in the group	In the 3months post-abortion day followed up whether the sexual life resumes and the contraceptive methods used each time. Urge women who use less effective contraception change to effective contraception methods. Correctly guide and adhere to use.Find out whether they are pregnant unintentionally again.Answer women’s questions, Telephone, clinic
**3Mths-6Mths**	Contraception: Encourage them to use effective contraceptions methods, and stick to them in case of unwanted pregnancy. Focus comments on the concerns of clients. Help people there and now behavior. Make frequent contact with people. Pills: Take estrogen and progestin pills and observe for side effects. Teach about pills and continue to replenish pills promptly when missing. Complications: if all else, see health care when not well. Recognize symptoms of complications and need to seek appropriate medical attention. Psychology: determine what you can and cannot do and search forassistance. Recognize personal strengths and use them to eliminate, avoid, reduce, and manage stress. Encourage: Answer correctly and give affirmative praise in the group discussion., Group Announcement, Speech in the group	In the 6months post-abortion day follow up about post abortion recovery,the menstrual condition, contraceptive measure use, Urge women who use less effective contraception change switch to efficient effective contraceptionmethods. Find out whether they are pregnant unintentionally again. Answer women’s questions, Telephone, clinic

Contraceptive methods were as follows [26–29]: Very effective and effective methods are divided into Effective (0–9 pregnancies per 100 women) and Moderately Effective (10–19 pregnancies per 100 women) by their effectiveness as commonly used; Less successful (at least 20 pregnancies per 100 women) Effective(IUDs, sub-dermal implant, estrogen, and progestin pills, condoms right used, tubal ligation), moderately effective (progestin pills, vaginal rings,trans-dermal patches, progestin subcutaneous and intramuscular inject-able, less effective (diaphragm, agents that kill sperm or impair sperm motility, fertility awareness, withdrawal).

The assessment was conducted six months following enrollment and intervention, with the period being "the present or past week." Call back six months after the abortion if there is an unexpected second pregnancy. Another was assessed using the self-rating anxiety scale (SAS) or self-assessment anxiety scale [30]. Each of the 20 questions has four possible answers. Sincerely, according to your circumstances throughout the past week, sum the scores from each question to get the initial score. Then, add the scores from the 20 things to get the raw score, multiply that by 1.25, and take the integer part to get the standard score (index score), which is the fifth, ninth, and so on. According to the findings of the Chinese norm calculations [31], the SAS standard point cutoff value is 50 points, which are used to score each item 13, 17, and 19. A typical score is below 50, followed by mild anxiety (50–59) and severe anxiety (60–69).

### Statistical analysis

The SPSS version 24.0 was used to compile and analyze the data. Counting data is represented by N(%), and measurements like age and the number of days till menstrual cessation time follow a normal distribution using the mean ± standard deviation ($\overline{x }$± S). Using an independent sample *t*-test and chi-square test, the data on age, marriage, occupation, education level, place of habitual residence, fertility status, number of induced or medical abortions, number of spontaneous abortions or induced labor, and abortion procedures were compared. Preoperative contraception, contraceptive methods used within 6 months after the abortion, and mental health data were compared between the two groups using the T-test and Chi-square.

## Results

Between the two groups of information that were provided, there were no statistically significant differences. The sociodemographic traits factors were as follows: age (years), menopausal days (days), married status (single/married/divorced), and occupation. Education, place of residence, number of births (natural or induced), and techniques of abortion used to end pregnancies (Table 2).

**Table 2:** Women characteristics among WeChat group and control group (*n* = 394)

Items	N ($\overline{x }$±S)		T- test/Chi-square	*P*
	WeChat Group	Control Group		
**Age** (Year)	30.12 ± 5.022	29.69 ± 5.261	0.825	0.410
Pregnancy Days	49.075 ± 9.392	50.306 ± 9.665	-1.281	0.201
Marital status			0.525	0.812
Single	51 (25.8)	56 (28.6)		
Married	140 (70.7)	132 (67.3)		
Divorce	7 (36.71)	8 (4.1)		
Occupation			2.082	0.724
Management personnel	77 (38.9)	70 (35.7)		
Professional personnel	22 (11.1)	20 (10.2)		
Self-employed	24 (12.1)	23 (11.7)		
unemployed	26 (13.1)	22 (11.2)		
others	49 (24.7)	61 (31.1)		
Education			6.163	0.105
Middle school graduates or lower	24 (12.1)	22 (11.2)		
High school graduate	39 (19.7)	30 (15.3)		
College graduate	62 (31.3)	48 (24.5)		
University graduate	73 (36.9)	96 (49.0)		
Living area			5.605	0.066
City or urban	168 (84.8)	176 (89.8)		
Rural	18 (9.1)	17(8.7)		
Field or Floating	12 (6.1)	3 (1.5)		
Number of Children born			5.564	0.062
0	55 (27.8)	68 (34.7)		
1	95 (48.0)	98 (50.0)		
≥ 2	48 (24.2)	30 (15.3)		
Pregnancy Termination(n)			3.090	0.215
1	151 (76.3)	155 (79.1)		
2	31 (15.7)	20 (10.2)		
≥ 3	16 (8.1)	21 (10.7)		
Number of Natural abortions or Induced Labor (n)			0.001	1.000
0	186 (93.9)	184 (93.9)		
≥ 1	12 (6.1)	12 (6.13)		
Abortion Method (n) Induced abortion	162 (81.80)	150 (76.50)	1.671	0.216
Oral medications abortion	36 (18.20)	46 (23.50)		

The main reason for the unintended pregnancy before intended abortion among the two groups was the use of less effective contraception [28, 29, 32], and there was no difference in the contraceptive methods taken by women before intended abortion. After the intervention, compared to the control group, the number of effective contraceptive measures increased noticeably in the WeChat group, and the changes were statistically significant (Table 3).

**Table 3:** Women's contraceptive methods among two groups before and after education

		WeChat Group	Control Group	*T-tes*t/ Chi-square	*P*
		N (%)			
Before Education	^a^Effective	4 (2.0)	3 (1.5)	0.602	0.747
	^a^Moderately Effective	40 (20.2)	45 (23.0)		
	^a^Less effective	154(77.8)	148 (75.5)		
After Education	^a^Effective	119 (63.0)	52 (28.6)	60.808	< 0.001
	^a^Moderately Effective	65 (34.4)	85 (46.7)		
	^a^Less effective	5 (2.6)	45 (24.7)		
	Chi-square	253.029	110.564		
	P	< 0.001	< 0.001		

^a^Effective (IUDs, sub-dermal implant, estrogen and progestin pills, condoms right used, tubal ligation), moderately effective (progestin pills, vaginal rings, trans-dermal patches, progestin inject-able (subcutaneous and intramuscular), less effective (diaphragm, agents that kill sperm or impair sperm motility, fertility awareness, withdrawal) [22]

In contrast, in the six months between the two groups' subsequent unintended pregnancies, the WeChat group had a re-pregnancy rate of 0, and the conventional PAC group had 2; one unintended pregnancy was brought on by the short-acting oral contraceptive pill not being taken on time after being missed, and the other by the lack of a contraceptive at the time (Table 4). Women who become pregnant unexpectedly experience distress and anxiety, which can have various psychological effects [8, 9]. Higher scores reflect greater worry. Before the education, 75 people in the conventional PAC group (37.9%) and 77 in the WeChat group (39.3%) experienced varying stress levels. Women's anxiety significantly decreased following instruction (45.63 ± 10.49 vs. 35.42 ± 5.06, 46.10 ± 9.10 vs.41.88 ± 8.64, *P* < 0.001), according to comparisons. In contrast, the WeChat group experienced a higher reduction in anxiety. Only 5 people in the WeChat group and 32 in the standard PAC group experienced mild anxiety following education when comparing phased needs-based education to traditional education. The two women's SAS scores are contrasted (Table 5).

**Table 4:** Comparison of unwanted pregnancy among two groups before and after education

		WeChat Group	Control Group	Chi-square
Whether unwanted pregnancy in 6 months	Yes(N)	0	2	0.1541
	No(N)	198	194

**Table 5:** Comparison of anxiety among two groups before and after education

	WeChat Group	Control Group	*T-test*/ Chi-square	P
	± S			
Before Education	45.63 ± 10.49	46.10 ± 9.10	0.474	0.636
After Education	35.42 ± 5.06	41.88 ± 8.64	9.045	< 0.001
Chi-square	12.27	4.372		
P	<0.001	< 0.001		

## Discussion

This study aims to apply the PAC service needs based on the WeChat platform to the post-abortion stage needs of women with early termination of pregnancy and to regularly push general science knowledge as group announcements based on this application effect. As a crucial component of current disease management, especially during the COVID-19 pandemic, people need to be isolated from each other with inconvenient travel. Health education is crucial in building good behavior patterns and reaching optimal health. Many domestic and international investigators have employed mobile phones to conduct patient follow-up visits and health education studies, all of which have produced unique outcomes [19, 33–37]. Establishing WeChat groups and placing women in an excellent postabortion clinical environment can help women interact quickly and get thorough information on abortion, post-abortion rehabilitation, and individualized treatment in the current era of the widely used mobile WeChat [13]. Studies have shown that those who experience less psychological discomfort value the most consoling sense of understanding because excessive psychological discomfort leads to worry. Including women in the group to deliver phased health education can also satisfy patients' needs and lessen psychological distress [38, 39].

The emotional gap between women and doctors is reduced through interactive contact in WeChat groups, increasing patients' trust and satisfaction with medical professionals and lowering anxiety [16, 20, 21, 34]. Patients' health behaviors are directly influenced by their cognitive needs, and by providing targeted health education, which helps improve the quality of education while helping patients change their beliefs to change their behavior, medical satisfaction is increased [4, 7].Unplanned pregnancies can be treated with induced abortion. However, the scope of family planning services is somewhat constrained in China due to the high rate of abortions performed following unplanned pregnancies. Our investigation of effective contraceptive measures coincided with the expert study [22, 26–29] that there are numerous issues with unplanned pregnancies due to the less effective contraceptive methods used, including lack of fertility awareness, cycle regularity, patient commitment to daily evaluation of symptoms (first morning temperature, cervical mucus consistency), and the patient's ability to avoid intercourse or ejaculation during the time of peak fertility [29], incorrect condom use, and withdrawal, and emergency contraceptive pills as an estrogen and progestin pills taken. These issues are the main causes of this contraceptive failure, and they lack ongoing guidance to acquire accurate and standardized knowledge of contraception and abortion [38]. However, because traditional health education is rather general, the health education method is not pertinence enough, and it is challenging to make patients have a deeper understanding of the disease under a single oral education, even though up to this point, the standardized PAC service provided by family planning workers has benefited many women [2–4, 7].

WeChat, the most popular method of exchanging information [13], offers the advantages of real-time, dynamic, and efficient engagement, giving patients access to more efficient, practical, and individualized health education. We can infer from the chat message of the WeChat engagement that the users' queries regarding postoperative recovery, medication, food, and menstruation transit require immediate attention. Contrarily, abortion causes patients' emotions to suffer greatly in addition to the physical harm it does to women [8, 19]. Additionally, to address the issues brought on by intended abortion and provide women with psychological treatment, we must conduct thorough, effective, and in-depth health education. At the same time, women who have had intended abortions can benefit from post-abortion contraception, rest, and diet through the interactive experience sharing between group members on Tencent [18].

This study gathered women's post-abortion knowledge needs by creating WeChat groups early on. The post-abortion demand model was then created through significant data computation by computer, and it was used to push health education demand information via WeChat group announcements and written educational programs. Based on the theoretical framework of IB (inquiry-based learning) [20, 21, 23], Include post-abortion women in WeChat groups. We use WeChat dialogue, regularly send health education information, one-on-one answers to questions, and consultation methods to explore the possibilities and advantages of WeChat health education for post-abortion women and show results. Based on various post-abortion times. In addition to monitoring how contraception is used in the experimental group and the control group throughout the 6-month trial, it is crucial to take steps to ensure that patients can quickly and effectively obtain the information they need, that they will readily cooperate with treatment, nursing, and additional facets of their care, and rehabilitation, and get the best possible outcomes in therapy. To analyze the success of women's health education through post-abortion follow-up and to provide ongoing services for abortion care and care for unwanted pregnancies, medical personnel conduct targeted health education for patients to ask questions through WeChat groups. The disadvantages of conventional education methods, such as lack of relevance and lack of understanding, are compensated for by this type of targeted health education approach of distributing pertinent educational resources to women in the form of group announcements so that patients can gradually get clear and persuasive health education, gain a personalized and in-depth understanding of their postabortion recovery, increase their awareness of contraception with effective methods, and prevent the physical and mental pain brought on by unwanted pregnancy. To ensure better and sufficient medical treatment, encourage postoperative recovery.

Additionally, the WeChat group is for family planning doctors and senior nurses to engage in personalized and targeted responses to the statements and experience sharing among group members according to the questions they raise, which fosters their confidence and hope in post-abortion rehabilitation and gives patients a sense of value and respect. Instead of absorbing much knowledge immediately, women prefer to learn about their preferences gradually. Needs-based patient education helps to determine the appropriate amount of information required to provide education based on patient needs. Their anxiety has decreased [20]. As a result, women pay more attention to their health and cooperate and comply with follow-up, fully demonstrating the satisfying outcome of applying the individualized postoperative health education needs model to carry out health education for patients. Additionally, it serves as a guide for post-abortion women who need ongoing care outside of hospitals.

First-trimester intended abortion women using the WeChat platform receive post-abortion phased needs-based education encouraging women to adopt effective contraceptive methods after an abortion actively, allowing the PAC continuation service to meet the needs of patients better, and prevent unintended pregnancy, reduce the rate of induced abortion and repeat abortion, live healthier lives, and ensure women's reproductive health and physical and mental well-being [34, 38, 40].

### The strenght and implications

This study supplemented the lack of offline, face-to-face health education through WeChat group health education based on the needs of women after abortion, especially during special periods such as the COVID-19 pandemic when people are inconvenient to travel and must be isolated from others. This kind of health education via WeChat can improve work efficiency so that women's problems can be consulted and answered in a timely manner, and women's satisfaction can be improved [19]. Participants with chronic conditions are more likely to use these services, and telemedicine use is rapidly changing. It is vital for healthcare providers to identify non-telemedicine users and their common characteristics. Monitoring patients' attitudes regarding telemedicine is essential in the future after the pandemic ends [37].

### Limitations

First-trimester abortion women should be guided to take appropriate contraceptive measures as soon as possible using post-abortion phased needs-based education on the WeChat platform, which can significantly increase the awareness rate of contraceptive methods and decrease anxiety among women with unplanned pregnancies. It is simply a single institution trial, though, because of the experiment's limited sample size. To improve the quality of health education continually due to incomplete representation, it is required to increase the study sample size in the future, perform personalized education for the actual understanding capacity of patients, and quickly evaluate the effect of education. Another limitation of the article is that it is only aimed at health education for groups who can use mobile phones to chat with WeChat. And Zung Psychosomatics. 1971 is just a generalized anxiety scale that could not judge women's anxiety results about unintended pregnancy, contraceptive measures, had intended abortion, or may not have any relevance to the questions around pregnancy.

## Conclusion

First-trimester intended abortion women who have had an intended abortion are given post-abortion phased needs-based education on the WeChat platform, based on IBL encouraging them to adopt effective contraceptives after having an intended abortion so that the PAC continuation service can better meet their current needs, which will help them prevent unwanted pregnancies effectively, lower the rate of induced abortion and repeat abortion, live healthier lives, and ensure their reproductive, physical, and mental health.

## Data Availability

The datasets used and/or analyzed during the current study are available from the corresponding author upon reasonable request.

## References

[1] Suh S (2020). What post-abortion care indicators don't measure: Global abortion politics and obstetric practice in Senegal. Soc Sci Med.

[2] Huber D (2019). Postabortion Care and the Voluntary Family Planning Component: Expanding Contraceptive Choices and Service Options. Glob Health Sci Pract.

[3] Jones RK, Jerman J (2017). Characteristics and circumstances of us women who obtain very early and second-trimester abortions. PLoS One.

[4] Baynes C, Yegon E, Lusiola G, Kahando R, Ngadaya E, Kahwa J (2019). Women's Satisfaction With and Perceptions of the Quality of Postabortion Care at Public-Sector Facilities in Mainland Tanzania and in Zanzibar. Glob Health Sci Pract.

[5] Stephens B, Mwandalima IJ, Samma A, Lyatuu J, Mimno K, Komwihangiro J (2019). Reducing Barriers to Postabortion Contraception: The Role of Expanding Coverage of Postabortion Care in Dar es Salaam. Tanzania Glob Health Sci Pract.

[6] Ohannessian A, Jamin C (2016). Contraception après interruption volontaire de grossesse [Post-abortion contraception]. J Gynecol Obstet Biol Reprod (Paris).

[7] Baynes C, Kahwa J, Lusiola G, Mwanga F, Bantambya J, Ngosso L, Hiza M (2019). What contraception do women use after experiencing complications from abortion? an analysis of cohort records of 18,688 postabortion care clients in Tanzania. BMC Womens Health.

[8] Horvath S, Schreiber CA (2017). Unintended Pregnancy, Induced Abortion, and Mental Health. Curr Psychiatry Rep.

[9] Farren J, Mitchell-Jones N, Verbakel JY, Timmerman D, Jalmbrant M, Bourne T. The psychological impact of early pregnancy loss. Hum Reprod Update. 2018 Nov 1;24(6):731–749. doi: 10.1093/humupd/dmy025. PMID: 30204882. Jones R, Jerman J, Ingerick M. Which Abortion Patients Have Had a Prior Abortion? Findings from the 2014 U.S. Abortion Patient Survey. J Womens Health (Larchmt). 2018 Jan;27(1):5However, postoperative complications caused by widespread miscarriage should not be ignored.8–63. doi: 10.1089/jwh.2017.6410. Epub 2017 Aug 23. PMID: 28832238; PMCID: PMC5771530. 10.1093/humupd/dmy025 30204882

[10] Potter JE, Stevenson AJ, Coleman-Minahan K, Hopkins K, White K, Baum SE, Grossman D (2019). Challenging unintended pregnancy as an indicator of reproductive autonomy. Contraception.

[11] Chaiken SR, Darney BG, Schenck M, Han L (2023). Public perceptions of abortion complications. Am J Obstet Gynecol.

[12] Ream E, Hughes AE, Cox A, Skarparis K, Richardson A, Pedersen VH, Wiseman T, Forbes A, Bryant A (2020). Telephone interventions for symptom management in adults with cancer. Cochrane Database Syst Rev.

[13] Zou Q, Zhang G, Liu Y (2018). Health Education Using Telephone and WeChat in Treatment of Symptomatic Uterine Myoma with High-Intensity Focused Ultrasound. Med Sci Monit Basic Res.

[14] Lee YJ, Kim ES, Choi JH, Lee KI, Park KS, Cho KB, Jang BK, Chung WJ, Hwang JS (2015). Impact of reinforced education by telephone and short message service on the quality of bowel preparation: a randomized controlled study. Endoscopy.

[15] Guruge GND, Rathnayake N, Abhayasinghe K (2022). Description of a telephone and Internet-based intervention to improve community responses to COVID-19 spread. J Health Popul Nutr.

[16] Corry M, Neenan K, Brabyn S, Sheaf G, Smith V (2019). Telephone interventions, delivered by healthcare professionals, for providing education and psychosocial support for informal caregivers of adults with diagnosed illnesses. Cochrane Database Syst Rev.

[17] Palmer MJ, Henschke N, Villanueva G, Maayan N, Bergman H, Glenton C, Lewin S, Fønhus MS, Tamrat T, Mehl GL, Free C. Targeted client communication via mobile devices for improving sexual and reproductive health. Cochrane Database Syst Rev. 2020 Jul 14;8(8):CD013680. doi: 10.1002/14651858.CD013680. PMID: 32779730; PMCID: PMC8409381. 10.1002/14651858.CD013680 PMC8409381 32779730

[18] Hill J, McGinn J, Cairns J, Free C, Smith C (2020). A Mobile Phone-Based Support Intervention to Increase Use of Postabortion Family Planning in Cambodia: Cost-Effectiveness Evaluation. JMIR Mhealth Uhealth.

[19] Qaderi K, Khodavirdilou R, Kalhor M, Behbahani BM, Keshavarz M, Bashtian MH, Dabir M, Irani M, Manouchehri E, Farahani MF, Mallah MA, Shamsabadi A (2023). Abortion services during the COVID-19 pandemic: a systematic review. Reprod Health.

[20] Gong L, Han J, Yan W, Qin Y. The effect of post-abortion care (PAC) on anxiety in women with spontaneous abortion based on MicroRNA-21 expression, cortisol level, and Fordyce happiness pattern. Cell Mol Biol (Noisy-le-grand). 2022;67(4):181–188. 10.14715/cmb/2021.67.4.20. PMID: 35809289. 10.14715/cmb/2021.67.4.20 35809289

[21] Wongkietkachorn A, Wongkietkachorn N, Rhunsiri P (2018). Preoperative Needs-Based Education to Reduce Anxiety, Increase Satisfaction, and Decrease Time Spent in Day Surgery: A Randomized Controlled Trial. World J Surg.

[22] Cwiak C (2020). Contraception for high risk patients. Semin Perinatol.

[23] Nadkarni A, Costa-Pinto R, Hensman T, Harman EV, Yanase F, Lister BG, Nickson CP, Thomas JS (2023). Evaluating an inquiry-based learning program. Adv Physiol Educ.

[24] Brown JA (2016). Evaluating the effectiveness of a practical inquiry-based learning bioinformatics module on undergraduate student engagement and applied skills. Biochem Mol Biol Educ.

[25] Noordzij M, Dekker FW, Zoccali C, Jager KJ (2011). Sample size calculations. Nephron Clin Pract.

[26] Teal S, Edelman A (2021). Contraception Selection, Effectiveness, and Adverse Effects: A Review. JAMA.

[27] Aztlan-James EA, McLemore M, Taylor D (2017). Multiple Unintended Pregnancies in U.S. Women: A Systematic Review. Womens Health Issues..

[28] Rocca ML, Palumbo AR, Visconti F, Di Carlo C (2021). Safety and benefits of contraceptives implants: a systematic review. Pharmaceuticals (Basel).

[29] Colquitt CW, Martin TS (2017). Contraceptive Methods. J Pharm Pract.

[30] Zung WW (1971). A rating instrument for anxiety disorders. Psychosomatics..

[31] ZhangFengxia.Study on Medical Health Status and Influencing Factors of Nurses in Grade 3A Hospitals.Peking.Chinese Acedemy of Medical Sciences Peking Union Medical College, 2023.

[32] Millimouno TM, Leno JP, Sidibé S, Bah OH, Delamou A, Hyjazi Y (2020). Assessment of Post-abortion Care Services in Two Health Facilities in Conakry. Guinea Afr J Reprod Health.

[33] Smith C, Gold J, Ngo TD, Sumpter C, Free C (2015). Mobile phone-based interventions for improving contraception use. Cochrane Database Syst Rev..

[34] Sudhinaraset M, Landrian A, Cotter SY, Golub G, Opot J, Seefeld CA, Phillips B, Ikiugu E (2022). Improving stigma and psychosocial outcomes among post-abortion Kenyan women attending private clinics: A randomized controlled trial of a person-centered mobile phone-based intervention. PLoS One.

[35] Veiga-Junior NN, Cavalari CA, Eugeni C, Kajiura BD, Stefano N, Baccaro LF (2020). Post-abortion contraception before hospital discharge after installation of a surveillance network in Brazil. Int J Gynaecol Obstet.

[36] Smith C, Ly S, Uk V, Warnock R, Free C (2017). Women's views and experiences of a mobile phone-based intervention to support post-abortion contraception in Cambodia. Reprod Health.

[37] Reicher S, Sela T, Toren O (2021). Using Telemedicine During the COVID-19 Pandemic: Attitudes of Adult Health Care Consumers in Israel. Front Public Health.

[38] Thorp JM, Hartmann KE, Shadigian E (2003). Long-term physical and psychological health consequences of induced abortion: review of the evidence. Obstet Gynecol Surv.

[39] Kercher EE (1991). Anxiety. Emerg Med Clin North Am.

[40] Coughtrey AE, Pistrang N (2018). The effectiveness of telephone-delivered psychological therapies for depression and anxiety: A systematic review. J Telemed Telecare..

