# Correction: Post-abortion needs-based education via the WeChat platform to lessen fear and encourage effective contraception: a post-abortion care service intervention-controlled trial

**DOI:** 10.1186/s12905-024-03033-y

**Published:** 2024-03-25

**Authors:** Danfeng Shi, Chenyin Liu, Lingna Huang, Xiao-Qian Chen

**Affiliations:** Fujian Provincial Maternal and Child Health Hospital, Fujian, Fuzhou, China

**Correction to: BMC Women’s Health (2024) 24:159**

10.1186/s12905-024-03004-3

Following publication of the original article [1], author’s name ‘Chenyin Liu’ was incorrectly written as ‘Chenying Liu’.

The original article has been corrected.
